# Mediastinal lymph node dissection and distal esophagectomy is not essential in early esophagogastric junction adenocarcinoma

**DOI:** 10.1186/s12957-016-1088-x

**Published:** 2017-01-18

**Authors:** In-Seob Lee, Ji-Yong Ahn, Jeong-Hwan Yook, Byung-Sik Kim

**Affiliations:** 1 Ulsan University College of Medicine, Asan Medical Center, Seoul, Korea; 2 Department of Surgery, Asan Medical Center, 88, Olympic-ro 43-gil, Songpa-gu, Seoul 05505 South Korea; 3 Department of Gastroenterology, Ulsan University College of Medicine, Ulsan, South Korea; 4 Department of Gastroenterology, Asan Medical Center, Seoul, South Korea

**Keywords:** Esophagogastric junction, Adenocarcinoma, Mediastinal lymphadenectomy, Total gastrectomy

## Abstract

**Background:**

Optimal extent of surgery remains controversial in types 2 and 3 adenocarcinoma of esophagogastric junction (AEG). We aimed to determine whether the extended procedure including mediastinal lymphadenectomy is essential in all patients with AEG by comparing prognosis and recurrence of proximal gastric adenocarcinoma based on total gastrectomy with intra-abdominal lymphadenectomy.

**Methods:**

The clinicopathologic characteristics of 672 patients (type 2: 90, type 3: 211, upper third of the stomach: 371 cases) who underwent curative total gastrectomy with lymphadenectomy between 2003 and 2009 were reviewed.

**Results:**

Recurrence was observed in 36.7, 16.1, and 16.1% of cases of type 2 AEG, type 3 AEG, and cancer of the upper third of the stomach, respectively. The 5-year disease-free survival rates were 62.6, 82.5, and 84.6%, respectively. Subgroup analysis revealed that in early cancers, there was no difference in survival between the groups (93.2 vs. 96.7 vs. 98.7%) but in advanced cancers, there was a difference (47.9 vs. 75.4 vs. 71.8%, *P* < 0.001). There was no survival difference in stage 1 (97.5 vs. 98.7 vs. 98.3%), but, in stage 2, type 2 AEG had a worse prognosis (41.9 vs. 92.1 vs. 83.0%). Types 2 and 3 advanced AEG had higher rates of locoregional recurrence, especially in the vicinity of the esophagojejunostomy and mediastinal lymph nodes compared to proximal gastric cancer.

**Conclusions:**

Total gastrectomy without mediastinal lymphadenectomy might produce favorable outcomes in early AEG and acquisition of a greater length of proximal margin, and removal of mediastinal lymph nodes might be helpful in advanced cancers.

**Electronic supplementary material:**

The online version of this article (doi:10.1186/s12957-016-1088-x) contains supplementary material, which is available to authorized users.

## Background

Adenocarcinoma of the esophagogastric junction (AEG) is associated with a poor prognosis, and its incidence has rapidly increased in Western countries [1–4]. However, in the Far East including Korea, although the incidence of adenocarcinoma in the upper third of the stomach is gradually increasing, data of AEG are lacking [5–8]. This may be due to the difficulty of defining the esophagogastric junction (EGJ), as well as the scarcity of the disease entity. AEG is defined as a tumor with an epicenter within 5 cm of the EGJ, and Siewert et al. classified EGJ cancer based on an endoscopic finding into type 1 (lower esophageal cancer), type 2 (true EGJ cancer), and type 3 (subcardial cancer), and this classification is still widely used [9].

While curative resection of the primary tumor and regional lymph nodes is the mainstay of the treatment of EGJ cancer, the extent of surgery is still controversial especially for types 2 and 3 AEG. Siewert et al. insisted that for patients with type 2 tumors, esophagectomy offered no advantage over extended gastrectomy if an R0 resection can be achieved [3]. On the other hand, several groups have reported the outcomes of surgical approaches including transthoracic or transhiatal esophagectomy, total gastrectomy, and proximal gastrectomy [10–12]. As each of these studies involved mixed patient groups undergoing a variety of surgical procedures and extents of lymphadenectomy, there are of limited use for evaluating the dependence of the prognosis and recurrence pattern of AEG on the specific procedure employed.

The aim of this study was to investigate the differences in the prognosis and pattern of recurrence of types 2 and 3 AEG compared with adenocarcinoma in the upper third of the stomach when employing the same operation as for proximal gastric cancer (total gastrectomy with intra-abdominal lymph node dissection), and to determine whether the extended procedure including mediastinal lymphadenectomy is essential in all AEG patients.

## Methods

### Patients

This retrospective study adhered to the guidelines established by the Declaration of Helsinki, and was approved by the institutional review board of Asan Medical Center. All patients who underwent curative total or extended total gastrectomy with D1+ or D2 lymphadenectomy based on gastric cancer for Siewert types 2 and 3 AEG at Asan Medical Center from 2003 to 2009 were reviewed. To compare the prognosis, patients who received the same operative procedure for adenocarcinoma in the upper third of the stomach during the same period were also investigated. All patients achieved pathologically cancer-free resection margin. Their medical records were reviewed to determine clinicopathologic characteristics, including age at operation, sex, tumor location, size, gross appearance, differentiation, Lauren’s classification, depth of invasion, number of metastatic and total harvested lymph nodes, lymphovascular and/or perineural invasion, adjuvant chemotherapy, and recurrence pattern. With regard to tumor location, the endoscopic findings in all of the patients were reviewed by the gastric surgeons and an experienced gastroenterologist. Patients that were excluded where those who had a suspicious metastatic lymph node in the mediastinum on preoperative examination, those who received prior chemo- or radio-therapy or those with synchronous malignancy, recurred or metastatic gastric cancers, tumors in the remnant stomach, and those with less than 15 harvested lymph nodes, and Borrmann type 4 tumours. From this initial pool, a total of 672 patients (301 AEG and 371 adenocarcinomas in the upper third of the stomach) were finally analyzed.

### Follow-up

Patients were regularly followed up and the study protocol included physical examination, blood tests, tumor markers, esophagogastroduodenoscopy, and abdominopelvic computed tomography. With regard to the site of recurrence, the first site at presentation was the one documented. Patterns of recurrence were classified into locoregional, distant metastasis, and both. Follow-up was continued until July 31, 2015 and the median period of follow-up was 60.8 months (range, 3.0 to 136.7 months).

### Histological evaluation

All surgical specimens were handled and examined according to the guidelines of the Japanese Gastric Cancer Association [13]. All histological slides were reviewed by experienced gastrointestinal pathologists.

Depth of invasion and nodal staging were determined according to the American Joint Committee on Cancer (AJCC) staging manual 7th edition [14]. The diagnosis of carcinoma was based on the modified Vienna classification, and the histological type was determined according to the World Health Organization (WHO) classification [15, 16].

### Statistical analysis

SPSS statistical software (version 12.0 for Windows, Chicago IL, USA) was used for all statistical analyses. The chi-squared test was used to assess the correlation between tumor location and sex, depth of invasion, differentiation, gross pattern, presence of lymphovascular/perineural invasion, and recurrence pattern. The Mann-Whitney *U* test was used to compare age, tumor size, and number of metastatic and harvested lymph nodes according to tumor location. Disease-free survival rate was calculated by the Kaplan-Meier method, and a multivariable Cox regression model was used to identify independent prognostic factors. Statistical significance was set at *P* < 0.05.

## Results

### Clinicopathologic findings of patients

During the period, a total of 672 patients with Siewert types 2 and 3 AEG and upper third of stomach cancer were analyzed. Their median age at operation was 60 years and, of them, 488 were men and 184 were women. Types 2 and 3 AEG and upper third cancers were found in 90, 211, and 371 cases. The median tumor size was 4.7 cm.

Three hundred ninety-five cases (58.8%) were advanced cancers. Histologically, 402 cases (59.8%) had undifferentiated histology, and the intestinal type in Lauren’s classification was the most dominant. The median number of harvested lymph nodes was 28 and stage 1 tumors were the greatest followed by stages 2 and 3. The median length of the proximal resection margin was 2.0 cm. Lymphovascular invasion and perineural invasion were identified in 30.8 and 26.0%, of the tumors, respectively. About half of the patients received adjuvant chemotherapy. The results are summarized in Table 1.

**Table 1:** Clinicopathologic characteristics of patients with adenocarcinoma of the EGJ and the upper third of the stomach

Variable	Value
Age in years, median (range)	60 (21–85)
Sex	
Male	488 (72.6%)
Female	184 (27.4%)
Tumor location	
Siewert 2	90 (13.4%)
Siewert 3	211 (31.4%)
Upper third	371 (55.2%)
Tumor size in cm, median (range)	4.7 (0.2–20.5)
Gross appearance	
I	13 (1.9%)
IIa/IIb/IIc	262 (39.0%)
III	6 (0.9%)
B1	18 (2.7%)
B2/B3	373 (55.5%)
Differentiation	
Differentiated	270 (40.2%)
Undifferentiated	402 (59.8%)
Lauren’s classification	
Intestinal	333 (49.6%)
Diffuse	265 (39.4%)
Mixed	72 (10.7%)
Not evaluated	2 (0.3%)
T stage	
T1	277 (41.2%)
T2	100 (14.9%)
T3	201 (29.9%)
T4a	80 (11.9%)
T4b	14 (2.1%)
Number of harvested lymph nodes, median (range)	28 (15–82)
N stage	
0	424 (63.0%)
1	98 (14.6%)
2	77 (11.5%)
3	73 (10.9%)
TNM Stage	
1	338 (50.3%)
2	179 (26.6%)
3	155 (23.1%)
Proximal resection margin, median (cm)	2.0 (0.1–10.5)
Lymphovascular invasion	
Identified	207 (30.8%)
Not identified	464 (69.1%)
Not evaluated	1 (0.1%)
Perineural invasion	
Identified	175 (26.0%)
Not identified	483 (71.9%)
Not evaluated	14 (2.1%)
Adjuvant chemotherapy	
Yes	312 (46.4%)
No	360 (53.6%)

### Comparison of clinicopathologic factors according to tumor location

Compared to upper third cancer, AEG showed elderly predominance, deeper invasion, and Borrmann type 2 or 3 on gross appearance. It was also strongly associated with differentiated tumor, intestinal type, and shorter proximal resection margin. Sex, tumor size, number of metastatic and harvested lymph nodes, and presence of lymphovascular and perineural invasion did not differ according to tumor location (Table 2).

**Table 2:** Comparison of clinicopathologic factors according to tumor location in patients with adenocarcinoma of the EGJ and the upper third of the stomach

Variables	Tumor location			*P* value
	Siewert 2	Siewert 3	Upper third	
Age in years, mean	63.1 (±11.5)	59.6 (±11.3)	56.5 (±11.7)	<0.001
Sex				=0.232
Male	71 (78.9%)	156 (73.9%)	261 (70.4%)	
Female	19 (21.1%)	55 (26.1%)	110 (29.6%)	
Tumor size in cm	4.36 (±2.33)	4.74 (±2.36)	4.78 (±2.71)	=0.365
Gross appearance				=0.001
I	4 (4.4%)	3 (1.4%)	6 (1.6%)	
IIa/IIb/IIc	26 (28.9%)	67 (31.8%)	169 (45.6%)	
III	0 (0.0%)	2 (0.9%)	4 (1.1%)	
B1	5 (5.6%)	8 (3.8%)	5 (1.3%)	
B2/B3	55 (61.1%)	131 (62.1%)	187 (50.4%)	
Differentiation				=0.003
Differentiated	49 (54.4%)	90 (42.7%)	131 (35.3%)	
Undifferentiated	41 (45.6%)	121 (57.3%)	240 (64.7%)	
Lauren’s classification^a^				=0.002
Intestinal	60 (67.4%)	109 (51.7%)	164 (44.3%)	
Diffuse	24 (27.0%)	77 (36.5%)	164 (44.3%)	
Mixed	5 (5.6%)	25 (11.8%)	42 (11.4%)	
T stage				=0.001
T1	30 (33.4%)	72 (34.1%)	175 (47.1%)	
T2	18 (20.0%)	36 (17.1%)	46 (12.4%)	
T3	25 (27.8%)	74 (35.1%)	102 (27.5%)	
T4a	15 (16.7%)	24 (11.4%)	41 (11.1%)	
T4b	2 (2.1%)	5 (2.3%)	7 (1.9%)	
No. of metastatic LNs	3.1 (±5.4)	2.1 (±3.9)	1.8 (±4.6)	=0.064
No. of harvested LNs	27.6 (±10.7)	29.5 (±12.1)	30.7 (±12.3)	=0.074
Stage				=0.015
1	40 (44.4%)	95 (45.0%)	203 (54.7%)	
2	21 (23.3%)	58 (27.5%)	100 (27.0%)	
3	29 (32.3%)	58 (27.5%)	68 (18.3%)	
Proximal margin, cm	0.75 (±0.66)	1.36 (±0.79)	3.41 (±1.95)	<0.001
Lymphovascular invasion^a^				=0.219
Present	34 (37.8%)	67 (31.9%)	106 (28.6%)	
Not identified	56 (62.2%)	143 (68.1%)	265 (71.4%)	
Perineural invasion^a^				=0.748
Present	26 (29.9%)	53 (26.5%)	96 (25.9%)	
Not identified	61 (70.1%)	147 (73.5%)	275 (74.1%)	

^a^These variables were missing from the pathology reports of some of the patients

### Comparison of prognostic factors and disease-free survival according to tumor location

A univariate analysis in both AEG and upper third adenocarcinoma revealed that T stage, N stage, and presence of lymphovascular and perineural invasion were significantly associated with disease-free survival. However, in a multivariable analysis, only T stage, N stage, and lymphovascular invasion remained prognostic factors (Additional file 1: Table S1).

**Fig. 1:**
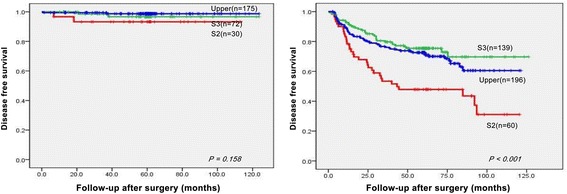
Disease-free survival curves in patients with adenocarcinoma of the EGJ and upper third of the stomach divided into early and advanced cancers

**Fig. 2:**
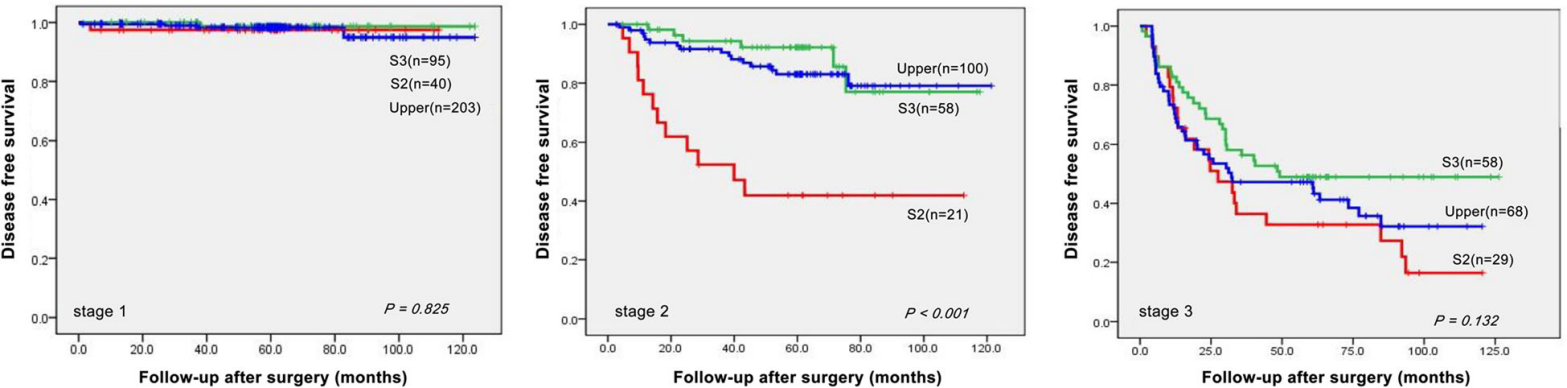
Disease-free survival curves in patients with adenocarcinoma of the EGJ and upper third of the stomach according to TNM stage

Recurrences were observed in 33 (36.7%), 34 (16.1%), and 60 (16.1%) cases of types 2 and 3 and upper third cancer, respectively. Kaplan-Meier curves were plotted to evaluate differences in disease-free survival according to tumor location. Type 2 AEG had a lower survival rate than type 3 tumors and those in the upper third of the stomach (*P* < 0.001). The 5-year disease-free survival rates in Siewert types 2 and 3 and upper third cancers were 62.6, 82.5, and 84.6%, respectively (Additional file 2: Figure S1). When disease-free survival was analyzed by depth of invasion, there were no differences in survival based on tumor location among early cancers (93.2 vs. 96.7 vs. 98.7%, *P =* 0.158). However, among advanced ones, there was statistically significant difference in survival (47.9 vs. 75.4 vs. 71.8%, *P* < 0.001) (Fig. 1). We also analyzed the survival according to stage based on the AJCC staging manual 7th edition. In stage 1, there were again no differences in survival (97.5 vs. 98.7 vs. 98.3%, *P =* 0.825). However, in stage 2, type 2 AEG had a lower survival rate than the other two groups (41.9 vs. 92.1 vs. 83.0%, *P <* 0.001). In stage 3, type 2 AEG appeared to have a worse prognosis but the effect was not statistically significant (32.8 vs. 48.9 vs. 45.2%, *P =* 0.132) (Fig. 2).

### Comparison of recurrence patterns according to tumor location

Distant metastasis including peritoneal seeding, paraaortic lymph node metastasis, and hematogenous spread was the most common routes of recurrence in all three types of cancers. However, type 2 and 3 AEGs had a higher incidence of locoregional recurrence than those in the upper third (*P =* 0.006). On the other hand, relapse at a distant site was more frequent in the tumors of the upper third of the stomach (Table 3). In type 2 and 3 AEGs, the most common locoregional recurrence sites were in the vicinity of esophagojejunostomy site (27.3 and 14.7% of all recurrences, respectively) and the mediastinal lymph nodes (6.1 and 2.9% of all recurrences, respectively), whereas they were in the celiac axis area in the upper third cancers. All locoregional recurrence sites were observed among advanced tumors and there was no local relapse among the early cancers.

**Table 3:** Comparison of recurrence sites according to tumor location in patients with adenocarcinoma of the EGJ and the upper third of the stomach

Recurrence Sites	Tumor location			*P* value
	Siewert 2	Siewert 3	Upper third	
Distant metastasis	20 (60.6%)	27 (79.4%)	55 (91.7%)	=0.006
Locoregional	5 (15.2%)	4 (11.8%)	3 (5.0%)	
Both	8 (24.2%)	3 (8.8%)	2 (3.3%)	
Near anastomosis	9	5	0	
Mediastinal lymph nodes	2	1	1	
Around celiac axis	1	0	4	
Others	1	1	0	

## Discussion

The prevalence of AEG in East Asian countries is much lower than that of tumors in the distal part of the stomach, and Siewert types 2 and 3 consists of the majority of AEGs. Moreover, the characteristics of AEG in the East have been reported to differ from in the West [17–19]. Although several previous studies have reported the surgical outcomes of AEG, most large-scaled studies were performed in the West and data from the East are relatively rare. In addition, the data on the recurrence patterns of AEG has a limitation that most studies reported the prognosis of several groups who underwent different extents of surgery [3, 20–22]. Despite some prospective randomized studies documenting the need for mediastinal lymph node dissection and transhiatal esophagectomy, the optimal surgical procedure for types 2 and 3 AEG has not been established [10, 12] and it is yet to be determined whether an extended procedure is essential for early AEGs. Until 2009, our department had a strategy for AEG of total gastrectomy with lymphadenectomy (plus, if necessary, combined resection of adjacent organs) similar to the strategy for primary proximal gastric cancer. This enabled us to analyze the prognosis and recurrence patterns of the two cancers based on the same operative procedure. To the best of our knowledge, this is the first study to investigate the prognosis of AEG compared to that of upper third cancer following total gastrectomy with D1+ or D2 lymph node dissection.

In many retrospective studies, AEGs were classified based not on endoscopic findings but on review of the postoperative specimens [1, 17, 18]. However, treatment with formalin could have caused specimens to shrink, and the size of tumors may have been overestimated or the tumors misclassified. To minimize such possibilities, we followed the proposal of Siewert’s classification based on endoscopic view, and classified the tumors by a review performed by experienced gastric surgeons and a gastroenterologist. In addition, a classification based on endoscopic findings would be more useful in clinical practice when identifying appropriate surgical strategies for treating different types of AEGs preoperatively.

The results of the present study differed in some epidemiologic and clinicopatholgic characteristics from other studies. Contrary to the previously reported greater proportion of males in AEG type 2 than type 3 [18, 23], there was no difference in sex ratio between the two types in this study. In addition, this study included a higher proportion of early cancers and smaller tumor size than other reports. This may be because of the national surveillance system for gastric cancer based on barium study or esophagogastroduodenoscopy that may detect cancers at earlier stage.

Radical resection with lymphadenectomy is the primary modality in the treatment of AEG, and currently transhiatal extended total gastrectomy is favored for type 2 AEG to remove probable metastatic lymph nodes in the mediastinum. Although postoperative complication rate of transhiatal resection is lower than that of the transthoracic approach, it has been reported to exceed 30% [10, 12]. This study demonstrated that total gastrectomy without mediastinal lymph node dissection could produce favorable oncologic outcomes in early AEG similar to those of primary upper third gastric cancer. There was no case of locoregional recurrence including the mediastinum, but two patients with T1N3 type 2 AEG experienced distant metastasis involving the liver and the paraaortic lymph nodes, respectively. Also, patients with stage 1 AEG had an excellent disease-free survival rate that did not differ from that of proximal gastric cancer. On the other hand, among the advanced cancers, type 2 AEG had a significantly worse prognosis than the other two types which had similar prognoses. Two randomized controlled studies reported a 5-year disease-free survival rates of 27.0~48.6% in a transhiatal group and 35.8~39.0% in a transthoracic group. Although it was not possible to compare these results because of the differences in the proportion of TNM stage between the groups, our survival rate of 47.9% in advanced type 2 AEG was better without mediastinal lymph node dissection.

In terms of recurrence, the tumors formed different patterns depending on their location. The proportion of locoregional recurrence was higher in type 2 and 3 AEGs than in upper third cancers, in which most recurrence involved distant metastasis. In type 2, in particular, about 40% of patients who experienced relapse had a local recurrence. This phenomenon may have resulted from inadequate local surgical control including insufficient proximal resection margins and absence of mediastinal lymph node dissection. Yuichi et al. reported recurrence rates of 44.4% in type 2 and 41.0% in type 3 AEG, which are markedly higher than in our results [24]. As the patients enrolled in that study did not receive adjuvant chemotherapy and the proportion of advanced cancers was higher, it is difficult to compare oncologic outcomes directly. However, it is remarkable that contrary to our findings, they reported that distant metastasis was dominant and local relapse was very rare. The two studies used similar abdominal surgery protocols based on total gastrectomy and abdominal lymphadenectomy including D2 dissection, and the presence of mediastinal lymphadenectomy plus esophagectomy might cause differences in the recurrence pattern. In addition, in both the Dutch and Japanese studies, although total recurrence rates were higher than in our case, the local recurrence was lower [10, 12]. Based on these findings, efforts to obtain an enough length of proximal resection margin and to remove mediastinal lymph nodes to minimize local relapse might be helpful in advanced type 2 and 3 AEGs. However, more research is required to prove the prognostic benefit of aggressive local treatment in AEG, because about half of the patients with local relapse also had distant metastases at the same time.

Although this study has limitations including a retrospective design and single institution data, its findings are valuable in as much they allow one to compare the prognoses of AEGs and primary proximal gastric carcinomas following the same operative procedure, and to compare their recurrence patterns. In addition, we propose that it is worth considering a tailored strategy that total gastrectomy without mediastinal lymphadenectomy might produce favorable outcome in early AEG and, in advanced AEG, the acquisition of a greater length of proximal margin with removal of mediastinal lymph nodes might be helpful. A better controlled prospective trial is required to prove the benefit of this strategy.

## Conclusions

Total gastrectomy with lymphadenectomy might produce favorable outcomes in early AEG and acquisition of a greater length of proximal margin with removal of mediastinal lymph nodes should be considered in advanced cancers.

## Additional files

Additional file 1: Table S1. Prognostic factors for disease-free survival of adenocarcinoma in of the EGJ and the upper third of the stomach in multivariable analysis. ^a^LVI refers to lymphovascular invasion. (DOC 30 kb)
Additional file 2: Figure S1. Disease-free survival curves in patients with adenocarcinoma of the EGJ and upper third. (TIF 4841 kb)

## Abbreviations

AEG: Adenocarcinoma of the esophagogastric junction
AJCC: American Joint Committee on Cancer
EGJ: Esophagogastric junction
