# Supplementary material for: Mediastinal lymph node dissection and distal esophagectomy is not essential in early esophagogastric junction adenocarcinoma
Source: World J Surg Oncol. 2017 Jan 18;15:28. doi: 10.1186/s12957-016-1088-x (PMC5242091; doi:10.1186/s12957-016-1088-x)
Supplement: Additional file 1: Table S1. — Prognostic factors for disease-free survival of adenocarcinoma in of the EGJ and the upper third of the stomach in multivariable analysis. aLVI refers to lymphovascular invasion. (DOC 30 kb) [file 12957_2016_1088_MOESM1_ESM.doc]

| **Variables** | **Adenocarcinoma of the EGJ** | | | **Adenocarcinoma in the upper third** | | |
| --- | --- | --- | --- | --- | --- | --- |
|  | **HR** | **95% CI** | ***P*-value** | **HR** | **95% CI** | ***P*-value** |
| T stage  T1  T2  T3  T4  N stage  N0  N1  N2  N3 | 1  1.577  3.533  3.736  1  3.317  8.482  10.950 | 0.376 – 6.608  0.988 – 12.636  0.933 – 14.957  1.169 – 9.413  3.128 – 23.000  3.751 – 31.968 | =0.046  <0.001 | 1  4.811  8.241  29.629  1  1.539  2.025  15.950 | 0.814 – 28.451  1.629 – 41.708  5.642 – 155.592  0.553 – 4.278  0.677 – 6.057  4.838 – 52.583 | <0.001  <0.001 |
| LVI^a^  Not identified  Present | 1  2.389 | 1.149 – 4.966 | =0.020 | 1  2.928 | 1.231 – 6.964 | =0.015 |
