# Supplementary figures and images for: Mediastinal lymph node dissection and distal esophagectomy is not essential in early esophagogastric junction adenocarcinoma
Source: World J Surg Oncol. 2017 Jan 18;15:28. doi: 10.1186/s12957-016-1088-x (PMC5242091; doi:10.1186/s12957-016-1088-x)

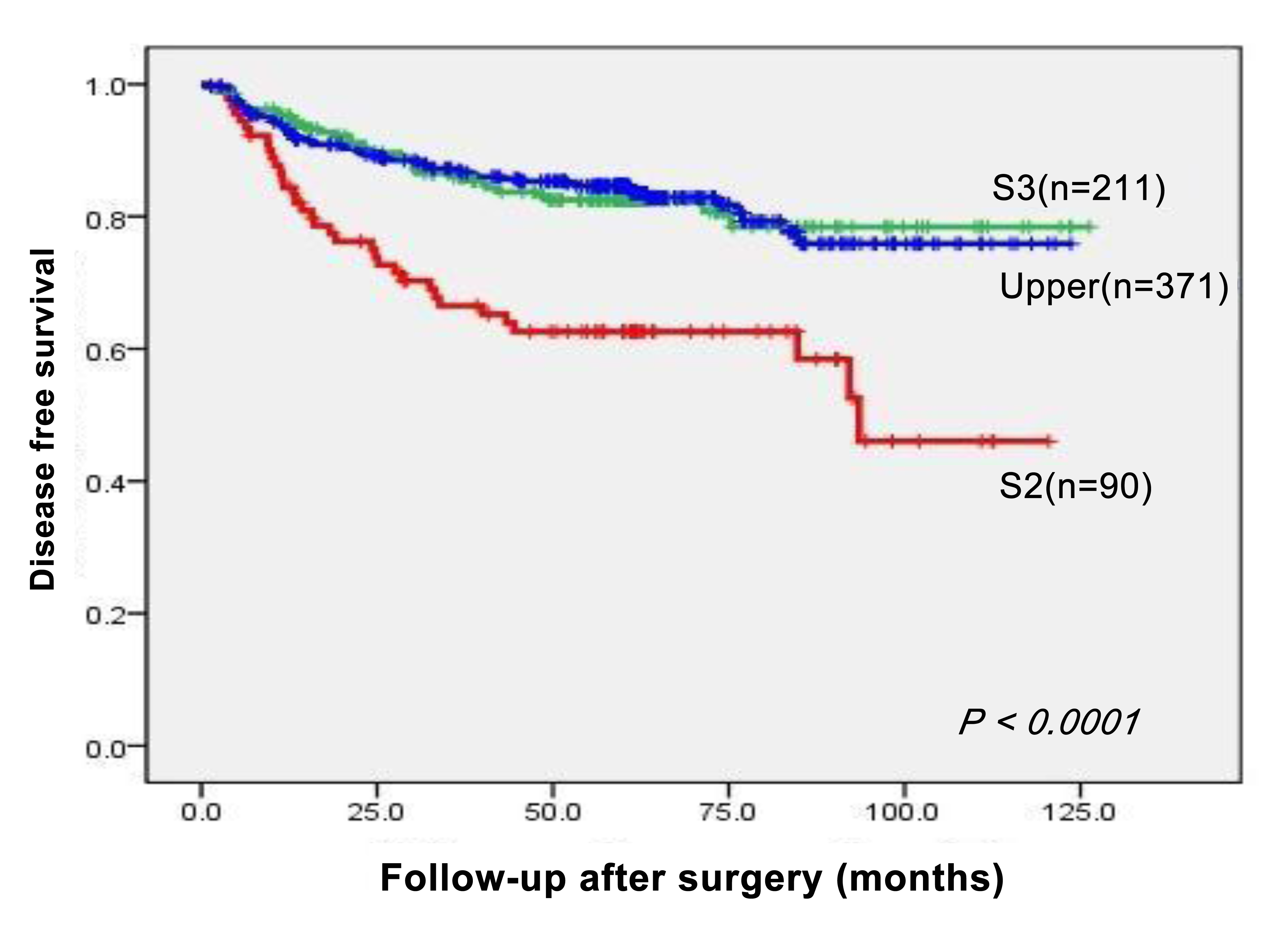

Supplement: Additional file 2: Figure S1. — Disease-free survival curves in patients with adenocarcinoma of the EGJ and upper third. (TIF 4841 kb) [file 12957_2016_1088_MOESM2_ESM.tif]
